# New York State Veterinary Medical Society

**Published:** 1897-01

**Authors:** Claude D. Morris

**Affiliations:** Secretary


### NEW YORK STATE VETERINARY MEDICAL SOCIETY.

(Continued from page 801 November number.)

Dr. John A. Bell, of Jefferson County, read a very interesting report of his county, showing that Watertown has a live board of health, and the members of the Society felt that the local board of health should be complimented for having as its sanitary officer so efficient a member as Dr. Bell. The report of Jefferson County is as follows:

"As committeeman for Jefferson County, N. Y., I am pleased to inform you that I live in a section of the county so far north that we are nearly free from contagious diseases. During the year 1895 we had three small outbreaks of anthrax in cattle, from which fifteen cows died, but so far this year, 1896, we have been entirely free from the disease.

"Regarding tuberculosis and the work done in that line in my section of the country I concluded to give you a synopsis of the workings of our board of health and the regulations governing the sale of milk, hoping that it will be of interest to some of you.

"Watertown is a city of 22,000 people, situated in Black River Valley, ten miles from Lake Ontario, and twenty-five miles from St. Lawrence River, in one of the best and most healthful dairying sections of the State. The climate in summer is mild, in winter severe, the thermometer falling sometimes to 15° below and occasionally to 25° below zero.

"In 1893, to preserve the cleanliness and purity of the milk, eliminate diseased cows and known impure and foul odors, the city board of health appointed me a veterinary inspector to supervise and inspect the supplying dairies and the vending of the milk to customers. The effect was largely experimental. There were then no regulations governing the supervision, and in consequence it had but little effect among the dairymen. The result was mainly instructive to the board in formulating the methods and supplying the remedies provided by the present system. In February, 1895, after a three months' careful study of the different features of the matter, with the aid of leading dairymen and milk-experts of the county, the board adopted the present regulations, which went into effect May 1, 1895, and appointed me its veterinary inspector.

"The regulations treat the subject of supervision under three heads: Duties of vendors, duties of producers, and duties of veterinary inspectors.

"*Duties of Vendors.* The vendor must obtain a license to sell, and the dairy must be examined and comply with the regulations before the license is issued. The license bears a serial number, which, with the word 'licensed' and the year, appear on each side of his wagon on plates furnished by the board; for example: '12, Licensed, 1896.' The license expires and must be renewed on the first day of each year. Renewals are governed by the same rules as new licenses. He must notify the health officer of the city of all cases of fever and contagious disease in his family and in those families where his milk is produced or cans washed, at once as they occur, and report on the first of each month to the inspector on blanks furnished by the board all such cases of sickness, whether he gave such notice to the health officer, what 'regular' and what 'emergency' dairies he sold from, and the number of cows added to the regular dairies during the month. The regulations are posted in all the dairy barns. The sale of milk at stores and stands must be under a permit naming the vendor furnishing it.

Cans must be washed without soap, salt being recommended; the last application being to scald with boiling water.

"*Duties of Producers.* Stables must be well ventilated with air-shafts from near the floor to the roof as foul-air outlets, well lighted and aired, kept whitewashed, clean, and dry, with trenches behind the cattle at least eight inches deep to receive the excrement; milkers to use no moisture on hands or teats while milking; dirt removed from the body of cow before milking.

"All milk must be thoroughly and slowly aërated in pure air by its exposure thereto in small quantities at a time, without ice or cold water, as soon as it comes from the cow and before cooling. In no case shall milk stand or be aërated in the stable, or in any building or place affected by stable or other foul or impure odors or air. The erection of small separate buildings for aërating in places surrounded with pure air is recommended.

"Cows must be fed on wholesome food and pure water, and be kept well cleaned. Milk must not be sold from any diseased cow.

"*Duties of Veterinary Inspector.* He is charged with the enforcement of the regulations. He must make two general inspections a year of all dairies and their surroundings, examining cattle as to health, cans, cattle, and stables as to care, and special inspections where and as need be. He examines dairies for and issues the licenses, and keeps the records belonging to the system; supervises the examination of the milk, reports monthly to the board upon all matters during the month, and makes an annual report at the end of the year.

"Any violation of the regulations is a misdemeanor, punishable as such. The board may also punish violations by revoking the license. The entire expense of the system is borne by the city, there being no law by which the milkmen can be made to share it.

"*Results of the System.* The adoption and operation of the system first met the determined opposition of some of the milkmen; a few were in favor and many were indifferent or sympathized with the opposition. Petitions were made and hearings had for its abandonment, as unnecessary, without precedent anywhere else, an innovation and wrongful interference with private right; that from experience and interest they were better fitted than the board to look after such matters. Opposition has now ceased and all have given it their willing support.

"We believe we are among the pioneers in supervising the sources of the public milk-supply, and that, from our own experience, such supervision is of the highest importance from a sanitary point of view.

"After the system had been in operation a little more than one year stables that were dark, ill-ventilated, covered with cobwebs and dirt, with poor or flat floors, without sufficient drop or trench to receive the excrement, have been given light, ventilation, cleanliness, new floors, with sufficient drop to keep the cattle clean, have been whitewashed, bedded, received better care generally; cattle better cared for and cleaned; the milk, which was often allowed to stand in the stable over night and not aërated at all, is now all properly aërated and kept entirely separate from the stables and stable odors.

"The publication of reports of the condition of stables and care of dairies and milk makes it necessary for the careless dealers to emulate the careful owners in order to hold their customers.

"About fifty dairies, numbering about 1000 cows, supply the city with milk and cream.

"The following improvements are taken from my annual report, made January 1, 1896, after the system had been in operation eight months: Stables whitewashed, 22; stables given better light, 18; stables given better ventilation, 17; stables given new floors, 11; stables given new ceilings, 7; stables given new linings, 2; new stables, 2; new aërating buildings, 8. All milk properly aërated in separate buildings.

"*Tuberculosis.* In 1895 the board made a bacteriological examination of the entire milk-supply, and discovered the presence of tuberculosis in the milk of 15 dairies, numbering 281 cows. The State board of health, upon request of the local board, administered the tuberculin-test to these 281 cows and found 8 cows afflicted with tuberculosis, which the State killed and paid for, all the animals killed showing evidence of the disease post-mortem.

"In two dairies the tuberculin-test disclosed no tubercular reaction where the bacteriological examination showed the presence of tuberculosis in the milk. The tuberculin-test showed that nearly 3 per cent. of the cows examined were tuberculous, and in addition marked 6 cows as suspicious cases.

"The board is fully awake to the danger of tuberculosis, and firmly believes in the efficacy of tuberculin to detect it. Its purpose is to examine for tuberculosis once a year, and also, in the interim, the newly added cows, which the vendors' monthly reports show will be about one-third of the dairies each year. Upon the failure of the State appropriation made to eradicate tuberculosis, the entire expense falls upon local boards, as is the case this year, as no law provides for killing or paying for the tuberculous cow, except when the State is making the test. We find this our greatest stumbling-block: What shall be done with the diseased cow? Her existence is a peril, and her use cannot be officially watched and prevented.

"The board believes that when bacteriological examination shows tuberculosis in the milk of a dairy, it has discovered a nuisance, a menace to health, and the dairymen should then be at expense of locating it and eradicating it, of abating the nuisance, as in the case of any nuisance, the work to be done by an expert under the approval of the local board; that when the tuberculin-test locates the disease in the cow the local board should have the power to kill the cow, the loss of the animal to be borne by the owner if post-mortem shows disease, and by the local board if not diseased; and that a law should be passed conferring such powers and imposing such duties, and we urge upon this Society that it take such steps as lie within its power toward securing proper legislation on this subject.

"*Meat-inspection.* No method of meat-inspection has yet been adopted, the expense having thus far stood in the way. The board favors a public slaughter-house where all animals should be killed under official supervision and where the rendering shall be done, and all meat brought into the city for sale, except that receiving government inspection, shall be examined. Slaughter-houses are now under the supervision of the board, but the results are unsatisfactory."

The discussion was taken part in by Drs. Berns, R. R. Bell, Crowforth, Cowie, W. L. Williams, and Huff.

The report of New York County, by Dr. O'Shea, is as follows:

"As County Secretary I beg leave to submit the report from New York County: Veterinary education has taken a decided step in our colleges in this city by an increase in the State requirements, it now being necessary for a student to pass the State Board of Regents' veterinary preliminary examination, and before receiving a certificate must gain twenty-four points. This requirement is to be further increased to forty-eight points, commencing with the session 1897–98. This means four years in a high school before entering college, and with the three sessions means a study of seven years before becoming qualified to practise.

"Our local board of health recently passed an ordinance requiring all milkmen to take out a license. They are also testing with tuberculin all the cows in the city, numbering about 3500; they are producing at present diphtheria antitoxin, mallein, tuberculin, and antitetanin.

"One fact I wish to bring before this Association is, that the meat- and milk-inspectors appointed by the health department are not veterinarians, and, in my mind, some action should be taken by this Association in the matter.

"The number of veterinarians at present employed in official positions in this city is as follows: City health department, 2; street-cleaning department, 5; fire department, 1; police department, 0. The United States department of agriculture employs 7 veterinarians as meat-inspectors at the various abattoirs.

"As to our experience with the use of tuberculin and mallein as diagnostic agents, we can only say that they have given us positive and gratifying results, and I can say that they are indispensable.

"My experience has been that we have not as yet an antitetanin of sufficient strength to cure tetanus, but as a preventive I recommend its use.

"The mortality in cattle from anthrax has been very materially reduced by the use of anthrax vaccine, although we have very little use for that agent in our large cities, especially in New York.

"The State inspection for the prevention and eradication of tuberculosis has been stopped, due to the lack of money. A bill presented during the last legislation asking for $300,000 for this purpose failed to pass. For the same reason the appraisers who were appointed to appraise and condemn glandered horses were dismissed, and at present no remuneration is given for glandered horses."

Discussion followed Dr. O'Shea's report, and a motion prevailed to draft a resolution under the seal of this Society, to be presented to the local boards of health, petitioning them to employ qualified veterinarians on the boards.

*Resolution.* The New York State Veterinary Medical Society, in annual convention in the city of Buffalo, September 4 and 5, 1896, do represent as follows: *Whereas*, the various positions of meat- and milk-inspectors of the State of New York are now being filled by men who are totally unqualified by education to fill the same; and, *whereas*, the public interests in these respects are suffering in consequence of such conditions; therefore, be it *Resolved*, that this Society respectfully petitions the said authorities to place in control of these important services none but graduates of recognized veterinary colleges.

Dr. Wilson Huff, county secretary for Oneida County, rendered an oral report, giving details of the sanitary requirements imposed by the board of health of the city of Rome on all dairymen who deliver milk in that city. The requirements are quite exacting. Dr. Huff is the sanitary inspector of dairies for the city. His work is giving the inhabitants of Rome pure, wholesome milk.

Oral reports were made of several other counties. They were consensual in the main that if the consumers of flesh- and dairy-products were properly protected against disease through the medium of these foods, it must of necessity be at the hands of the veterinarian.

A motion prevailed, signed by Drs. O'Shea and Baker, to amend the By-laws, Article VIII., Chapter I., relative to annual dues, to read as follows: "The dues shall be three dollars per annum, payable in advance." It was ordered spread upon the minutes to take its regular course at the next annual meeting.

Also a motion prevailed, signed by H. Sutterby and W. L. Baker, to amend Article VIII., Chapter I., of the By-laws, to read as follows: "The application of a candidate shall be accompanied with the initiation fee of five dollars and the first annual dues of two dollars, and if elected the money so received shall be appropriated to the use of the Society. The elect, after signing the By-laws and Constitution, and upon the presentation of the Secretary-Treasurer's receipt for the amount named, shall be presented by the Secretary with a copy of the By-laws and certificate of membership. If the candidate be rejected, the money so received shall be returned to him."

The Board of Censors reported favorably the following applicants: F. C. Gernside, Mt. Morris; Harry D. Gill, New York; G. C. Kister, Holly; John T. Liddle, West Shelby; F. D. Markham, Utica; J. O. Moore, Wilson; Charles John Mulvey, Moore's; J. A. McCrank, Plattsburg; W. H. Pendry, Brooklyn; Romanzo Perkins, Hardy's; W. L. Williams, Ithaca; Mark D. Williams, Middleport.

A motion was carried to suspend Section 3 of Article V., relative to the election of candidates, and that the candidates presented be received into the Society by unanimous consent.

A motion was carried to levy an assessment on the members of sufficient size conforming with the By-laws to meet the obligations of the Society, and that half of the requisite amount shall be collected during the years 1896–1897, but members elected at the annual meeting of 1896 shall be exempted from said assessment. Meeting adjourned.

SECOND DAY, SATURDAY, SEPTEMBER 5TH, 9 A.M.

Meeting called to order by the Secretary. Dr. R. R. Bell was asked to preside, Dr. Hinkley being absent. Unfinished and miscellaneous business was taken up. Letters of regret were presented from Drs. Latourell, of Yonkers; Giffen, of New York City; L. McLean, of Brooklyn; and J. W. Darby, of Fort Plain.

The resignation of Dr. J. H. Ferster, of New York City, was offered. A motion prevailed to lay the matter on the table, with instructions directing the Secretary to communicate with Dr. Ferster to ascertain the cause and report at the next annual meeting.

Dr. Crowforth, of Lockport, presented grievances relative to a paper read by him at the last annual meeting of the Society, on the ground that his paper did not receive professional recognition after the same had been solicited by the editor of the JOURNAL OF COMPARATIVE MEDICINE AND VETERINARY ARCHIVES. The JOURNAL, after receiving Dr. Crowforth's paper, refused to publish it, accusing the author of plagiarism. Drs. George Berns and John Wende held to the opinions, which were coincident, that Dr. Crowforth's complaint was out of order. The chair, Dr. R. R. Bell, overruled these opinions, on the ground that Dr. Crowforth was a member of the Society; that he had been asked by its President to prepare and read a paper, he had complied, which made his paper Association property, and he was entitled to be heard. Dr. Crowforth asked to be allowed to re-read his paper, that the members present might judge of its merit. The chair concured. After the paper had been read the Society ordered it laid on the table.

It was moved to proceed with the regular order on the programme: Reading of professional papers was in order. Dr. Thomas Giffen, of New York, was called to read a paper on "Veterinary Education;" absent. Dr. George H. Berns was called to read a paper on "Navicular Disease in Horses," but Dr. Berns asked that his paper be held over for the next regular meeting, and the chair so ordered. Dr. J. P. Thompson, of Niagara Falls, was called to present his paper on "Jurisprudence;" absent. Dr. Wilson Huff, of Rome, read his paper, "Bacteria in Milk," which was listened to with interest and heartily received. The chair requested Dr. Pendry, of Brooklyn, to open the discussion. In doing so the doctor gave some of the details of dairy-inspection in Kings County as required by the health department of Brooklyn. He spoke earnestly of the needs of a pure and uncontaminated milk-supply for our cities and of the many insidious conditions which expose it to infection, and thought the Society should use its best endeavors to procure such legislation as would establish systematic inspection, quarantine, and fair indemnity throughout the State for all cattle condemned and slaughtered as suffering with contagious and infectious diseases. Dr. W. L. Baker, of Cortland, related some of the conditions surrounding the dairy interests of Cortland County, and called special attention to one dairy near the city of Cortland that was selling its product in that city until a clinical inspection revealed that the herd was tuberculous; the health board then refused the milk, but it is now being sold in New York City. Dr. John Wende spoke of the character of some of the infantile diseases in Buffalo, and stated that 75 per cent. of the deaths of children from enteric troubles was due largely to unclean nursing-bottles with long nursing-tubes, and believed there would be much less enteric trouble among children if mothers and nurses would use the utmost caution in cleaning them. The discussion on Dr. Huff's paper was instructive and interesting. The doctor was given a vote of thanks for his very able paper. A paper entitled "Veterinarians as Sanitarians" was read by C. D. Morris, after which the meeting adjourned for lunch.

### AFTERNOON SESSION, SATURDAY, SEPTEMBER 5, 1896.

Meeting called to order at 1.30 P.M., Dr. R. R. Bell in the chair.

A communication was read which had been sent to the Secretary from the Cosmos Club, of Washington, D. C., soliciting funds to aid in the erection of a monument to the memory of M. Pasteur in Paris. The Society directed the Secretary to open a subscription, asking for an amount between twenty-five and fifty cents from all the members, to give a receipt for and keep a record of the same.

The chair appointed Drs. H. Sutterby and H. S. Wende as Auditing Committee to verify the financial accounts of the Secretary for the current year.

After a brief discussion relative to the place for the next annual meeting, the Society decided to go to Syracuse.

The Auditing Committee reported Secretary-Treasurer's accounts correct.

Thus ended one of the most profitable and enjoyable meetings this Society has ever held, from the view-point of numbers and interest.

CLAUDE D. MORRIS,
Secretary.

